# Effectiveness of Duct Excision Procedures in Detecting Preneoplastic and Malignant Lesions in Pathological Nipple Discharge: A Retrospective Cohort Study

**DOI:** 10.1155/tbj/2467046

**Published:** 2025-12-15

**Authors:** Batuhan Ata, Volkan Karadağ, Kenan Çetin

**Affiliations:** ^1^ Department of General Surgery, Faculty of Medicine, Çanakkale Onsekiz Mart University, Çanakkale, Turkey, comu.edu.tr; ^2^ Department of General Surgery, Kartal Dr. Lutfi Kirdar Training and Research Hospital, University of Health Sciences, Istanbul, Turkey, akdeniz.edu.tr

**Keywords:** breast carcinoma, duct excision, intraductal lesions, microductectomy, nipple discharge

## Abstract

**Background and Aims:**

Nipple discharge ranges from benign to pathological, indicating inflammation or epithelial proliferation. In 5%–28% of cases, pathological nipple discharge (PND) may indicate breast carcinoma. Our objective was to evaluate the detection rates of malignant and high‐risk lesions (HRL) in patients undergoing major duct excision (MDE) and microductectomy for diagnostic purposes due to PND and to assess the need for re‐excision in malignancies.

**Method:**

Patients diagnosed with PND between October 2015 and December 2023 underwent duct excision procedures after physical, imaging, and histopathological examinations, if necessary. Patients with malignancies detected by histopathological evaluation underwent oncological procedures and were excluded from the study.

**Results:**

Among 118 patients, 80 underwent microductectomy and 38 underwent MDE. Intraductal lesions (ILs) were detected in 62% of cases, with higher detection rates in the microductectomy group (69% vs. 47%, *p* < 0.03). Of these lesions, 23 were classified as HRL (24% in the microductectomy group vs. 11% in the MDE group, *p* = 0.09). Malignancy was detected in 16 patients (13.6%), with a higher rate in the MDE group (18% vs. 11%, *p* = 0.3). Five patients required re‐excision for clear surgical margins, with no significant difference between the groups (microductectomy: *n* = 2; MDE: *n* = 3, *p* = 0.3).

**Conclusion:**

The malignancy detection rate was slightly higher in the MDE group; however, this difference was not statistically significant. Similarly, there was no significant difference in the need for re‐excision. Microductectomy, which preserves lactation function, may be preferred for premenopausal individuals or those considering future pregnancies when clinical presentation supports single‐duct involvement. The differing distribution of IL and HRL between procedures reflects the pathology associated with their respective clinical indications rather than a difference in diagnostic performance.

## 1. Introduction

Nipple discharge is the third most common reason for seeking consultation at breast clinics [1]. When the discharge originates from a single duct in the breast, is bloody or serous in nature, occurs spontaneously (without manipulation), and is not related to lactation or pregnancy, it is termed pathological nipple discharge (PND) [2–4]. While the most common cause of PND is a single papilloma (a benign intraductal lesion [IL]), 5%–28% of cases may indicate breast carcinoma, highlighting the importance of ruling out malignancy [5–7]. Although traditional breast imaging modalities (mammography, ultrasonography, magnetic resonance imaging, and galactography) are widely used to diagnose PND, they may not be sufficient as stand‐alone diagnostic tools and primarily provide diagnostic insight rather than therapeutic intervention [8–11]. Therefore, after excluding malignancy through physical examination, conventional imaging, and, if necessary, histopathological examination, duct excision procedures are used to determine the cause of the discharge, exclude any underlying malignancy, and treat the discharge [9, 12]. In our breast clinic, we prefer microductectomy, especially for women of reproductive age and those planning to become pregnant in the near future, as it offers both diagnostic and therapeutic benefits and does not compromise breastfeeding function. For those not planning to become pregnant and those in menopause, we prefer the more traditional major duct excision (MDE).

The aim of this study was to compare the lesion detection rates of both methods in patients with PND who underwent microductectomy or MDE at our breast clinic.

## 2. Materials and Methods

### 2.1. Study Design and Patients

This study was designed as a retrospective cohort study and included female patients aged 18–90 who underwent surgery for PND at Kartal Training and Research Hospital and Çanakkale Onsekiz Mart University Faculty of Medicine, General Surgery Clinics, between October 2015 and October 2020 and between November 2020 and December 2023. Data were collected prospectively during and after surgery, with results presented retrospectively.

All procedures performed in studies involving human participants were conducted in accordance with the ethical standards of the institutional and/or national research committee, as well as the 1964 Helsinki Declaration and its later amendments or comparable ethical standards. The study protocol was approved by the Ethics Committee of Dr. Lutfi Kırdar Kartal Research and Training Hospital, affiliated with the University of Health Sciences (approval number: 514/95/3), and by the Clinical Research Ethics Committee of Çanakkale Onsekiz Mart University (approval number: 2025‐148). Written informed consent was obtained from all individual participants included in the study.

The patients were categorized into two groups based on the procedure performed: the first group comprised those who underwent microductectomy and the second group included those who underwent MDE. Patient characteristics (such as age, menopausal status, affected side, and number of pathological criteria), benign and preneoplastic lesion detection rates, efficiency in malignancy detection, and re‐excision requirements were compared between the two groups.

### 2.2. Exclusion Criteria

**Figure 1:**
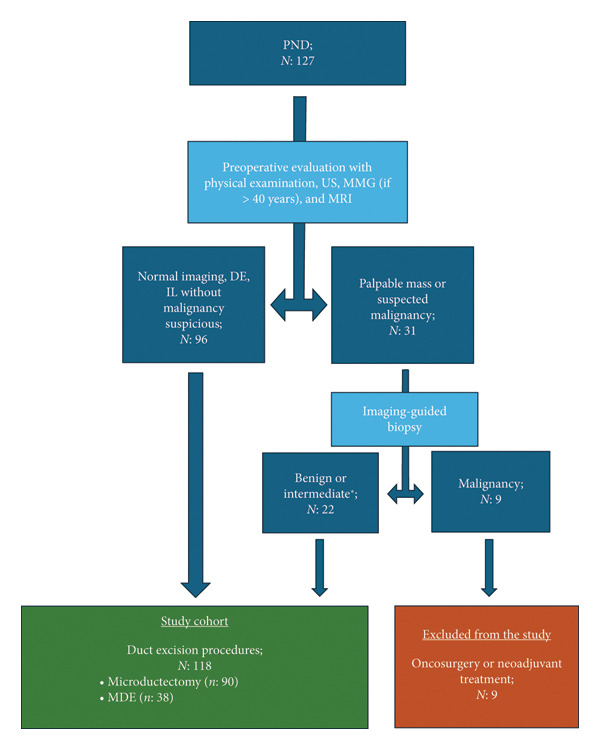
Flowchart of the study. PND, pathological nipple discharge; US, ultrasound; MMG, mammography; MRI, magnetic resonance imaging; DE, ductal ectasia; IL, intraductal lesion; MDE, major duct excision. ∗Papillary lesion and papillary neoplasia.

Patients with a palpable mass lesion and confirmed malignancy in their biopsies (Figure 1), high suspicion of malignancy based on imaging findings (Breast Imaging Reporting and Data System [BIRADS] 4b, 4c, or 5) and confirmed malignancy in their biopsies, and a history of previous breast cancer in the same breast were excluded from the study cohort.

### 2.3. Technical Details of Procedures

**Figure 2:**
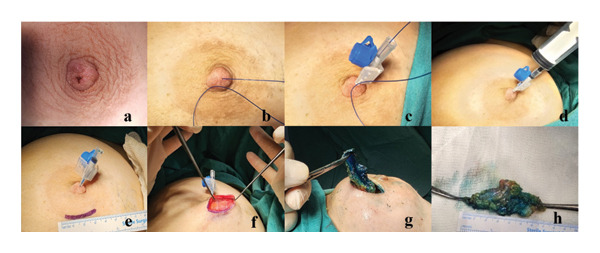
Microductectomy procedure: marking (a–d) and excision of the pathological duct with a periareolar incision (e–h).

The microductectomy procedure begins under either local or general anesthesia, following preparation of the surgical field and application of sterile draping. Microductectomy was performed under local anesthesia with 1% lidocaine (5–10 mL) or general anesthesia depending on patient preference and anesthetic risk. The symptomatic breast is gently manipulated to induce discharge (Figure 2(a)), allowing the identification of the pathological duct. Once the duct responsible for the discharge is located, a 2/0 Prolene suture thread is inserted into it (Figure 2(b)). The Prolene acts as a guide, facilitating duct intubation with a 22‐gauge blue Angiocath (Figure 2(c)). Subsequently, 1% methylene blue (1 mL) is injected through the Angiocath to stain the ductal lumen (Figure 2(d)). The blue‐stained duct is then dissected up to 6–7 cm distal from the subareolar region through a minimal periareolar incision (Figures 2(e), 2(f), 2(g), and 2(h)). Intraoperative bleeding was controlled with monopolar cautery, and no intraoperative complications or postoperative infections occurred. Following excision of the stained duct, the skin was closed subcutaneously with absorbable sutures.

In the MDE procedure, all major ducts leading to the nipple were excised through an inferior periareolar incision.

### 2.4. Statistics

Statistical analyses were performed using IBM SPSS Statistics for Windows, Version 20.0 (IBM Corp., Armonk, NY, USA). Parametric variables were compared using Student’s *t*‐test, and categorical variables were analyzed with the chi‐square test. Cases with missing imaging or cytology data were excluded from the relevant subgroup analyses; no imputation was applied. To identify independent predictors of lesion detection, a multivariate logistic regression analysis was performed, including age, menopausal status, imaging findings (presence of any abnormality), and type of surgery (microductectomy vs. MDE) as covariates. Odds ratios (ORs) with 95% confidence intervals (CIs) were calculated. All statistical tests were two‐sided, and a *p* value < 0.05 was considered statistically significant.

## 3. Results

### 3.1. Demographic and Clinical Findings

A total of 118 patients were included in the study. Among them, microductectomy (Group Mic) was performed in 80 patients (67%), while MDE (Group MDE) was performed in 38 patients (33%). The average age of both groups was similar (Group Mic: 48 ± 12.6 years [range: 25–81] and Group MDE: 47.3 ± 14.1 years [range: 27–67], *p* = 0.8), as were the premenopausal ratios (Group Mic: *n* = 42 [52%] and Group MDE: *n* = 18 [52.6%], *p* = 0.7) (Table 1).

**Table 1:** Comparison of demographic and preoperative findings.

Parameters	Group Mic (*n* = 80)	Group MDE (*n* = 38)	*p* values
Age, mean ± SD (range)	48 ± 12.6 (25–81)	47.3 ± 14.1 (27–67)	0.8
Premenopausal, *n* (%)	42 (52)	18 (52.6)	0.7
The side of PND (right), *n* (%)	43 (53.8)	20 (52.6)	0.9
Discharge type, *n* (%)	71‐9	37‐1	0.2
Spontaneously/with provocation	(89‐11)	(97‐3)	
Number of discharge ducts, *n* (%)	79/1	34‐4	0.03
Single–multiple	(99‐1)	(89‐11)	
Discharge color, *n* (%)	36‐31‐13	13‐8‐17	0.007
Bloody–serous–others; *n* (%)	(45‐39‐16)	(34‐21‐45)	
MMG findings, *n* (%)	22‐54‐4	8‐28‐2	0.7
NA–BIRADS 0, 1, 2–3, 4a	(27.5‐67.5‐5)	(21‐74‐5)	
US findings, *n* (%)	41‐39	17‐21	0.5
Normal–DE ± IL	(51‐49)	(45‐55)	
MRI findings, *n* (%)	12‐41‐27	9‐16‐13	0.5
NA–normal–IL	(15‐51‐34)	(24‐42‐34)	
Cytology findings, *n* (%)	10‐30‐39‐1	9‐18‐9‐2	0.04
NA–NS–PLS–atypia	(12‐38‐49‐1)	(24‐48‐23‐5)	

Abbreviations: DE, ductal ectasia; IL, intraductal lesion; MDE, major duct excision; MMG, mammography; MRI, magnetic resonance imaging; NA, not applied; NS, nonspecific findings; PLS, papillomatous lesion suspicious cytology; PND, pathological nipple discharge; SD, standard deviation; US, ultrasound.

There were no significant differences between the groups in terms of discharge side or type (spontaneous vs. provoked). However, a significant difference was observed regarding discharge color (bloody/serous/other) and number of ducts with discharge (single vs. multiple) (*p* = 0.007 and *p* = 0.03, respectively) (Table 1).

While imaging findings did not differ significantly between the groups (*p* > 0.05), cytological results showed a significant difference (*p* = 0.04) (Table 1).

The mean operative time was 30 ± 8 min for microductectomy and 20 ± 5 min for MDE.

### 3.2. Pathology Findings

ILs were more frequently detected in the microductectomy group compared to the MDE group (68.8% vs. 47.4%, *p* = 0.03). Although there was no significant difference between the two groups regarding the detection of malignant or high‐risk lesions (HRL), atypia‐free papillomas were detected significantly more often in the microductectomy group (45.1% vs. 18.4%, *p* = 0.008) (Table 2).

**Table 2:** Comparison of pathology results of the patients.

Pathological findings	Group Mic (*n* = 80)	Group MDE (*n* = 38)	*p* values
IL detected, *n* (%) (SP, MP, ADH, LCIS, DCIS, IC)	55 (68.8)	18 (47.4)	0.03
Papillomatous lesion/s without atypia, *n* (%)	36 (45.1)	7 (18.4)	0.008
Papillomatous lesion/s with atypia, *n* (%)	10 (12.5)	4 (10)	0.7
DCIS, *n* (%)	6 (7)	3 (7.9)	0.9
Invasive ductal carcinoma, *n* (%)	3 (3.8)	4 (10.5)	0.1
IL not detected, *n* (%) (DE, PM, DH, APM)	25 (31.2)	20 (52.6)	
Ductal ectasia–periductal mastitis (%)	20 (25)	18 (47)	0.02
Ductal hyperplasia–apocrine metaplasia (%)	5 (6.3)	2 (5.3)	0.8

Abbreviations: ADH, atypical ductal hyperplasia; APM, apocrine metaplasia; DCIS, ductal carcinoma in situ; DE, ductal ectasia; DH, ductal hyperplasia; IC, invasive breast cancer; IL, intraductal lesion; LCIS, lobular carcinoma in situ; MDE, major duct excision; MP, multiple intraductal papilloma; PM, periductal mastitis; SP, single intraductal papilloma.

In total, HRL and malignant lesions were detected in 19.5% and 13.6% of all patients, respectively. The malignancy detection rate was higher in the MDE group (18.4% vs. 11.3%), whereas HRL detection was higher in the microductectomy group (23.8% vs. 10.5%); however, these differences were not statistically significant (*p* = 0.3 and *p* = 0.09, respectively) (Table 3).

**Table 3:** Comparison of high‐risk lesions, malignancy, and re‐excision rates.

Pathological categories	Group Mic (*n* = 80)	Group MDE (*n* = 38)	*p* values
HRL, *n* (%) (MP, ADH, LCIS)	19 (23.8)	4 (10.5)	0.09
Malignancy, *n* (%) (DCIS, IC)	9 (11.3)	7 (18.4)	0.3
Re‐excision	2 (22.2)	3 (42.8)	0.3

Abbreviations: ADH, atypical ductal hyperplasia; DCIS, ductal carcinoma in situ; HRL, high‐risk lesion; IC, invasive breast cancer; LCIS, lobular carcinoma in situ; MDE, major duct excision; MP, multiple intraductal papilloma.

### 3.3. Multivariate Analysis

In the multivariate model adjusting for age, menopausal status, and imaging findings, both the presence of abnormal imaging and the type of surgery were significant independent predictors of lesion detection. Patients with any abnormal imaging finding had a markedly higher likelihood of lesion detection (OR = 7.11; 95% CI 2.89–17.52; *p* < 0.001). Similarly, microductectomy was independently associated with an increased detection rate compared with MDE (OR = 3.42; 95% CI 1.34–8.77; *p* = 0.01). Age and menopausal status did not significantly affect lesion detection (*p* > 0.05) (Table 4).

**Table 4:** Multivariate logistic regression analysis for lesion detection.

	OR	95% CI for OR	*p* value
Age	1.03	0.97–1.09	0.39
Menopausal status (postmenopausal)	1.44	0.32–6.40	0.64
Any abnormal imaging finding	7.11	2.89–17.52	< 0.001
Type of surgery (microductectomy vs. MDE)	3.42	1.34–8.77	0.01

### 3.4. Clinical Outcomes

A patient who underwent microductectomy and was diagnosed with ductal ectasia experienced a recurrence of nipple discharge during the first postoperative month. Reoperation with MDE revealed ductal ectasia and a foreign body reaction. All patients achieved symptomatic relief, and no postoperative complications occurred. Re‐excision was required to obtain adequate margins in five of the 16 malignancy cases (microductectomy: *n* = 2; MDE: *n* = 3), with no significant difference between the groups (*p* = 0.3) (Table 3). Sentinel lymph node biopsy was performed in four patients diagnosed with invasive carcinoma (microductectomy: *n* = 2; MDE: *n* = 2).

## 4. Discussion

PND requires thorough evaluation to differentiate between benign and malignant causes. Initial assessments include a detailed history, physical examination, mammography, and ultrasonography, with image‐guided biopsy performed when indicated. While most cases are due to benign conditions such as intraductal papilloma or duct ectasia, a smaller but clinically relevant proportion results from malignancy. The diagnostic pathway becomes particularly challenging in patients with no detectable lesions on initial imaging.

In our study, microductectomy identified benign pathology in the majority of patients, with intraductal papilloma being the most common cause of discharge, consistent with existing literature [13–16]. The overall malignancy incidence was 13.6%, including both invasive cancer (6%) and ductal carcinoma in situ (DCIS) (7.6%). In the subset of patients with negative clinical exams and initial imaging, 3% had invasive carcinoma and 10% had DCIS, which aligns with other studies [14, 15]. All patients with negative clinical exams, cytology, and initial imaging required further evaluation through duct excision procedures. This contrasts with studies recommending close follow‐up for patients with negative initial imaging, as our findings suggest that 14% of malignancies would have been missed, potentially delaying definitive treatment.

Our results provide insight into the diagnostic value of microductectomy and MDE in evaluating preneoplastic and malignant lesions in PND. Both procedures contributed significantly to the detection of IL, HRL, and malignancies, consistent with prior literature supporting the role of duct excision in PND management [1, 9, 12–15].

To the best of our knowledge, the only study comparing microductectomy with MDE in patients with PND is by Sharma et al. [12]. They found that the detection rates of malignancy and DCIS were significantly higher with MDE but did not compare IL and HRL detection rates. In our study, we found an overall IL detection rate of 62%. Microductectomy demonstrated a higher proportion of IL (69%) compared with MDE (47%); however, this difference is expected, given that microductectomy is primarily performed for single‐duct discharge, a clinical presentation in which intraductal papilloma and other IL‐associated lesions are inherently more common. Similarly, the HRL detection rate was 27% in the microductectomy group and 13% in the MDE group; this pattern also reflects indication‐driven patient selection rather than a diagnostic advantage of one technique over the other.

In terms of malignancy detection, our study found a slightly higher rate in the MDE group (18%) compared to that in the microductectomy group (11%), although this difference was not statistically significant. This pattern is consistent with the clinical presentation typically leading to MDE, as multiduct discharge is more often associated with malignant etiologies than single‐duct discharge. These findings align with the observations of Lustig et al. [1], supporting the view that each procedure reflects the pathology profile of its corresponding indication rather than differences in diagnostic capability.

The need for re‐excision to obtain clear surgical margins was observed in 5 (31.3%) patients (microductectomy group: *n* = 2; MDE group: *n* = 3) out of 16 malignancy cases. The lack of significant difference in re‐excision rates between the groups suggests that both procedures are comparably effective in achieving clear margins in patients with PND in whom malignancy has been excluded by physical examination, conventional imaging, cytology, and, where necessary, histopathological examination.

Microductectomy, which preserves lactation function, may be a suitable option for premenopausal women or those considering future pregnancies when the clinical presentation supports single‐duct disease. In our cohort, the distribution of IL and HRL detected after microductectomy reflects the underlying pathology typically associated with single‐duct nipple discharge, rather than a procedural advantage in detecting these lesions. This underscores the importance of selecting the surgical approach based on clinical indication while preserving breast function when appropriate.

This study is limited by its retrospective design, which may introduce selection bias, and by the heterogeneity between two centers and time periods. Although the cohort size was adequate for initial analysis, the relatively small sample size may limit the generalizability of our findings. Prospective, multicenter studies with larger cohorts are warranted to validate these results and further clarify how clinical presentation and indication influence pathological outcomes in patients undergoing duct excision. Further investigations focusing on long‐term outcomes—such as recurrence rates and overall survival—would provide additional insight. Moreover, the incorporation of adjunctive imaging modalities, including contrast‐enhanced mammography and molecular breast imaging, may improve preoperative diagnostics and optimize patient selection for duct excision procedures.

## 5. Conclusion

Conventional imaging modalities and cytology are insufficient to rule out malignancy in patients presenting with PND; therefore, duct excision remains essential to avoid missing a potential cancer diagnosis. Our study demonstrates that both microductectomy and MDE are effective in detecting IL, HRL, and malignancies in patients with PND, yet the two procedures serve different clinical indications. Microductectomy is predominantly performed in single‐duct discharge, where intraductal papilloma and other HRL are more commonly encountered, whereas MDE is preferred in multiduct discharge, with distinct underlying pathological conditions. Although the malignancy rate is slightly higher in the MDE group, this difference is not statistically significant, and the higher rate of IL in the microductectomy group reflects indication‐based patient selection rather than procedural superiority. Preservation of lactation function with microductectomy may offer a clinical advantage in appropriately selected premenopausal women or those planning future pregnancies; nonetheless, both procedures provide comparable efficacy in terms of surgical margin safety. Further studies are warranted to validate these findings and to refine indication‐based decision‐making for optimal surgical management.

## Ethics Statement

All procedures performed in studies involving human participants were conducted in accordance with the ethical standards of the institutional and/or national research committee, as well as the 1964 Helsinki Declaration and its later amendments or comparable ethical standards. The study protocol was approved by the Ethics Committee of Dr. Lutfi Kırdar Kartal Research and Training Hospital, affiliated with the University of Health Sciences (approval number: 514/95/3), and by the Clinical Research Ethics Committee of Çanakkale Onsekiz Mart University (approval number: 2025‐148). Written informed consent was obtained from all individual participants included in the study.

## Consent

Please see the Ethics Statement.

## Disclosure

Kenan Çetin affirms that this manuscript is an honest, accurate, and transparent account of the study being reported; that no important aspects of the study have been omitted; and that any discrepancies from the study as planned have been explained. The funding source had no role in study design, data collection, analysis, interpretation, or decision to submit the manuscript. All authors have read and approved the final version of the manuscript. Kenan Çetin had full access to all of the data in this study and takes complete responsibility for the integrity of the data and the accuracy of the data analysis.

## Conflicts of Interest

The authors declare no conflicts of interest.

## Author Contributions

Concept: Batuhan Ata and Kenan Çetin. Design: Batuhan Ata, Volkan Karadağ, and Kenan Çetin. Data collection and/or processing: Kenan Çetin, Batuhan Ata, and Volkan Karadağ. Analysis and/or interpretation: Kenan Çetin and Batuhan Ata. Literature search: Batuhan Ata, Volkan Karadağ, and Kenan Çetin. Writing: Batuhan Ata, Volkan Karadağ, and Kenan Çetin. Critical review: Kenan Çetin.

## Funding

The authors received no financial support for the research, authorship, and/or publication of this article.

## Data Availability

The data supporting the findings of this study are available from the corresponding author upon reasonable request.
